# Association analysis of indel variants and gene expression identifies *MDM4* as a novel locus for skeletal muscle hypertrophy and power athlete status

**DOI:** 10.1113/EP091992

**Published:** 2024-07-23

**Authors:** Hasan H. Kazan, Anıl Kasakolu, Seyrani Koncagul, Mehmet A. Ergun, George John, Rinat I. Sultanov, Andrey V. Zhelankin, Ekaterina A. Semenova, Rinat A. Yusupov, Nikolay A. Kulemin, Andrey K. Larin, Edward V. Generozov, Celal Bulgay, Ildus I. Ahmetov

**Affiliations:** ^1^ Department of Medical Biology, Gulhane Faculty of Medicine University of Health Sciences Ankara Türkiye; ^2^ Graduate School of Natural and Applied Sciences Ankara University Ankara Türkiye; ^3^ Department of Medical Genetics, Faculty of Medicine Gazi University Ankara Türkiye; ^4^ Transform Specialist Medical Centre Dubai UAE; ^5^ Department of Molecular Biology and Genetics Lopukhin Federal Research and Clinical Center of Physical‐Chemical Medicine of Federal Medical Biological Agency Moscow Russia; ^6^ Research Institute of Physical Culture and Sport Volga Region State University of Physical Culture, Sport and Tourism Kazan Russia; ^7^ Department of Physical Culture and Sport Kazan National Research Technical University Named after A.N. Tupolev‐KAI Kazan Russia; ^8^ Sports Science Faculty Bingol University Bingol Türkiye; ^9^ Sports Genetics Laboratory St Petersburg Research Institute of Physical Culture St Petersburg Russia; ^10^ Laboratory of Genetics of Aging and Longevity Kazan State Medical University Kazan Russia; ^11^ Research Institute for Sport and Exercise Sciences Liverpool John Moores University Liverpool UK

**Keywords:** athlete, genetics, muscle fibre

## Abstract

Insertions and deletions (indels) are the second most common type of variation in the human genome. However, limited data on their associations with exercise‐related phenotypes have been documented. The aim of the present study was to examine the association between 18,370 indel variants and power athlete status, followed by additional studies in 357,246 individuals. In the discovery phase, the D allele of the *MDM4* gene rs35493922 I/D polymorphism was over‐represented in power athletes compared with control subjects (*P *= 7.8 × 10^−9^) and endurance athletes (*P *= 0.0012). These findings were replicated in independent cohorts, showing a higher D allele frequency in power athletes compared with control subjects (*P *= 0.016) and endurance athletes (*P *= 0.031). Furthermore, the D allele was positively associated (*P *= 0.0013) with greater fat‐free mass in the UK Biobank. *MDM4* encodes a protein that inhibits the activity of p53, which induces muscle fibre atrophy. Accordingly, we found that *MDM4* expression was significantly higher in the vastus lateralis of power athletes compared with endurance athletes (*P *= 0.0009) and was positively correlated with the percentage of fast‐twitch muscle fibres (*P *= 0.0062) and the relative area occupied by fast‐twitch muscle fibres (*P *= 0.0086). The association between *MDM4* gene expression and an increased proportion of fast‐twitch muscle fibres was confirmed in two additional cohorts. Finally, we found that the *MDM4* DD genotype was associated with increased *MDM4* gene expression in vastus lateralis and greater cross‐sectional area of fast‐twitch muscle fibres. In conclusion, *MDM4* is suggested to be a potential regulator of muscle fibre specification and size, with its indel variant being associated with power athlete status.

## Abstract (highlights)

- **What is the central question of this study?**
  Which indel variants are functional and associated with sport‐ and exercise‐related traits?
- **What is the main finding and its importance?**
  Out of 18,370 tested indels, the *MDM4* gene rs35493922 I/D polymorphism was found to be the functional variant (affecting gene expression) and the most significant, with the deletion allele showing associations with power athlete status, fat‐free mass and cross‐sectional area of fast‐twitch muscle fibres. Furthermore, the expression of *MDM4* was positively correlated with the percentage of fast‐twitch muscle fibres and the relative area occupied by fast‐twitch muscle fibres.

## 1 INTRODUCTION

Athletic performance depends on many aspects, including genetically determined biochemical, anthropometric, physiological and psychological parameters, in addition to training, nutrition, lifestyle, culture and other environmental factors (Bayraktar et al., 2023; Wang et al., 2016). Depending on the sporting discipline, on average, 66% of the variance in athletic status is explained by additive genetic factors. The remaining variance is attributable to non‐shared environmental factors (De Moor et al., 2007). Despite the relatively high heritability of athletic status, the search for genetic variants contributing to a predisposition to succeed in certain types of sport has been challenging.

More than 400 million rare or common DNA variants have been found in humans across multiple populations (Taliun et al., 2021). The DNA variants include single nucleotide polymorphisms, indels (insertions/deletions of different lengths), copy number variations, duplications, inversions and translocations. With genotyping becoming widely available, a large number of genetic case–control studies evaluating candidate gene variants in athletic cohorts have been published, reporting largely unconfirmed associations with elite athletic status (Ahmetov et al., 2022).

So far, >250 DNA variants have been reported to be associated with athletic status (Semenova, Hall et al., 2023). Most of these variants belong to the class of single nucleotide polymorphisms, whereas seven genetic markers have been identified as indels. Indel variants are defined by the addition or loss of one or more nucleotides in a DNA sequence. As the second most common type of variation in the human genome, indels have been linked to many phenotypes. In sports genetics, the most studied indels are polymorphisms located in the angiotensin I converting enzyme (*ACE*; insertion or deletion of 287 bp; Ahmetov et al., 2008; Bulğay et al., 2020) and bradykinin receptor B2 (*BDKRB2*; insertion or deletion of 9 bp; Sawczuk et al., 2013; Williams et al., 2004) genes.

The less studied indels in relationship to exercise‐ and sport‐related traits include polymorphisms located in the protein phosphatase 3 regulatory subunit B, alpha (*PPP3R1*; 5I/5D; Akhmetov et al., 2008), GLIS family zinc finger 3 (*GLIS3*; rs34706136 TG/T; Guilherme, Semenova, Borisov et al., 2022), JunD proto‐oncogene, AP‐1 transcription factor subunit (*JUND*; rs10686842 TAAA/T), CDK5 regulatory subunit associated protein 1 like 1 (*CDKAL1*; rs745771286 G/GA) and protein kinase domain containing, cytoplasmic (*PKDCC*; rs3035165 T/TTA) genes (Semenova, Pranckevičienė et al., 2023).

It should be noted that most exercise‐ and sport‐related indels have been discovered using the PCR method and the candidate gene approach. Other approaches, including genome‐wide association (Al‐Khelaifi et al., 2020; Bojarczuk et al., 2022; Pickering et al., 2019; Semenova et al., 2022; Yang et al., 2023), exome‐wide association (Bulgay et al., 2023) and whole genome sequencing (Boulygina et al., 2020) studies, have proved to be extremely successful in uncovering genetic associations with exercise‐ and sport‐related phenotypes, such as maximal oxygen uptake, sprinting performance, reaction time, muscle fibre composition, exercise‐induced fat loss and fat‐free (muscle) mass.

The strength of the genotyping methods lies in their unbiased nature, not being constrained by the genomic structure or pre‐existing trait knowledge, unlike candidate gene studies, which rely on prior information to pinpoint loci associated with the desired trait. Unlike the previous study by Bulgay et al. (2023), the present research broadens its investigative horizon to encompass gene‐expression analysis, with a particular focus on indels and power‐related traits (lean mass, percentage of fast‐twitch muscle fibres and muscle fibre size) using a larger control group and replication cohorts. By leveraging results from a case–control study in the Turkish population, this study incorporates data from the UK Biobank and human muscle biopsies to reinforce its conclusions. We hypothesize that an exome‐wide association study on indels could reveal new genetic loci linked to power‐related traits.

The main purpose of this study was to examine the association between 18,370 indel variants and power athlete status, followed by morphological and gene‐expression analyses and bioinformatic studies. To achieve this, genomic, transcriptomic, morphological and bioinformatic analyses were performed on human participants.

## 2 MATERIALS AND METHODS

### 2.1 Ethical approval

The study was approved by the Non‐Interventional Clinical Research Ethics Committees of Gazi University (approval number 09; 5 April 2021) and the Ethics Committee of the Federal Research and Clinical Centre of Physical–Chemical Medicine of the Federal Medical and Biological Agency of Russia (approval number 2017/04; 4 July 2017). The FUSION [Database of Genotypes and Phenotypes (dbGaP) Study Accession: phs001048.v2.p1] and GTEx (dbGaP accession number phs000424.vN.pN) parts of the study were approved by local Ethics Committees, as previously described (GTEx Consortium, 2020; Taylor et al., 2019). Written informed consent was obtained from each participant. The study complied with the *Declaration of Helsinki* and ethical standards for sport and exercise science research.

### 2.2 Participants

The first case–control study involved 60 Turkish athletes [31 power athletes: 11 females and 20 males; 29 endurance athletes: 10 females and 19 males; mean (SD): age 25.1 (4.8) years; height 175.0 (7.9) cm; body mass 72.5 (22.4) kg; sport experience 9.4 (4.8) years] licensed in different clubs and affiliated with the Turkish Athletics Federation. The power group included athletes whose events demand predominantly anaerobic energy production. The athletes in this group (*n* = 31) were 100−400 m runners (*n* = 9), jumpers (*n* = 3) and throwers (*n* = 19). The endurance athlete group (*n* = 29) included athletes competing in long‐distance events demanding predominantly aerobic energy production. This group included 3000 m (*n* = 12), 5000 m (*n* = 5), 10,000 m (*n* = 4) and marathon (*n* = 8) runners. All athletes were ranked nationally in the top 10 in their sports discipline and had participated in international competitions such as the Olympic Games, European Championships, Universiade, Mediterranean Games and Balkan Championship. Accordingly, athletes were classified as highly elite (*n* = 16; prize winners of international competitions) and elite (*n* = 44; participants in international competitions). None of the athletes tested positive for doping. The number of Turkish control subjects was 20, and they were healthy, unrelated citizens of Turkish descent without any competitive sports experience. Moreover, sequence data from 557 healthy individuals from the Turkish Genome Project (https://tgd.tuseb.gov.tr/en/; accessed on 15 April 2024) was also used for the verification of frequencies of candidate variants obtained after statistical association analyses, because this database allows only singular variant search. All the athletes and control subjects were of Caucasian ancestry.

The second case–control study involved 477 Russian athletes [267 males and 210 females; age 26.5 (5.5) years], of whom 159 were elite sprinters (30 100−400 m runners, 40 500−1000 m speed skaters, 35 sprint cyclists, 25 50−100 m swimmers and 29 200−500 m kayakers or canoeists), 89 were strength athletes (56 weightlifters and 33 powerlifters), 57 were speed–strength athletes (3 arm‐wrestlers, 15 alpine skiers, 4 decathletes, 10 throwers, 22 jumpers and 3 skeleton racers) and 172 were endurance athletes (32 biathletes, 5 long‐distance cyclists, 90 cross‐country skiers, 3 marathon runners, 13 long‐distance swimmers, 7 race walkers and 22 triathletes). Overall, sprinters (*n* = 159), speed–strength athletes (*n* = 57) and strength athletes (*n* = 89) were categorized as power athletes (*n* = 305). Athletes were classified as highly elite (*n* = 256; prize winners of international competitions), elite (*n* = 195; participants in international competitions) and sub‐elite (*n* = 26; participants in regional competitions). None of the athletes tested positive for doping. Control subjects were 206 healthy and unrelated citizens of Russia without any competitive sport experience [27 females and 179 males; age 41.6 (6.9) years].

The muscle biopsy study of athletes (analysis of *MDM4* gene expression in vastus lateralis) involved 10 sub‐elite power [four powerlifters, four weightlifters, one decathlete and one taekwondo athlete; age 30.1 (7.4) years; height 178.2 (6.7) cm; body mass 85.6 (12.4) kg] and 12 sub‐elite endurance [eight long‐distance runners, three triathletes and one cross‐country skier; age 34.9 (10.1) years; height 181.9 (6.3) cm; mean body mass 75.6 (10.2) kg] male athletes of European descent (Russians). The muscle biopsy study of 291 sedentary individuals (analysis of *MDM4* gene expression in vastus lateralis) involved 166 men [age 59.5 (8.1) years, height 176.7 (6.7) cm; body mass 87.3 (15.1) kg] and 125 women [age 60.3 (8.1) years, height 162.8 (5.6) cm, body mass 71.8 (9.8) kg] of European descent from the FUSION study, as previously described (Taylor et al., 2019). The muscle biopsy study of 791 individuals from the GTEx project (analysis of *MDM4* gene expression in gastrocnemius) involved 535 males (age 20–79 years) and 256 females (age 20–79 years) of European descent, as previously described (GTEx Consortium, 2020).

### 2.3 Whole exome sequencing

For the case–control study on Turkish participants, the genotyping was performed by whole exome sequencing. For this aim, total genomic DNA was isolated from the peripheral blood of the participants using the DNeasy Blood and Tissue Kit (Qiagen, Germany) according to the supplier's instructions. The quality of the isolated DNA was verified using 1% agarose gel electrophoresis, and concentration was determined by NanoDrop (NanoDrop 1000 Spectrophotometer; Thermo Scientific, USA). Whole exome sequencing was performed after library preparation by the Twist Human Comprehensive Exome Panel (Twist Biosciences, USA). In brief, DNA was fragmented enzymatically, size selection was carried out and hybridization was applied using Twist Hybridisation probes and Dynabeads MyOne Streptavidin T1 (Invitrogen, USA), and the library was enriched by PCR. The concentration and size of the libraries were determined, and libraries were sequenced using Illumina NextSeq500 according to the manufacturer's standard protocol. Raw data were processed by the Genome Analysis Toolkit (GATK; Van der Auwera et al., 2013) HaplotypeCaller program to obtain Binary Alignment Map (BAM) files and subsequently produce an output Variant Call Format (VCF) file via the GRCh38/hg38 reference genome (Figures S1‐S4). Variants were annotated by ANNOVAR (Wang et al., 2010).

### 2.4 Genotyping

Molecular genetic analysis was performed with DNA samples obtained from leucocytes (4 mL of venous blood) of Russian athletes and control subjects. DNA extraction and purification were performed using a commercial kit according to the manufacturer's instructions (Technoclon, Moscow, Russia). Genotyping of single nucleotide polymorphisms was performed using microarray technology (Illumina, San Diego, CA, USA) with HumanOmni1‐Quad and HumanOmniExpress BeadChips (Illumina), as previously described (Kikuchi et al., 2022).

### 2.5 Gene‐expression analysis

Prior to obtaining a muscle biopsy of the vastus lateralis of the left leg (in the morning), athletes (*n* = 22) were asked not to train for 1 day to analyse their gene‐expression profiles in the resting state. The RNeasy Mini Fibrous Tissue Kit (Qiagen, Hilden, Germany) was used to isolate RNA from 22 muscle tissue samples of athletes, as previously described (Zhelankin et al., 2023). The RNA concentration was measured using the Qubit spectrophotometer (Thermo Fisher Scientific, Waltham, MA, USA). RNA quality was assessed using the BioAnalyzer electrophoresis system and BioAnalyzer RNA Nano assay (Agilent Technologies, Santa Clara, CA, USA). The RNA integrity number (RIN) was calculated for each RNA sample. Only RNA samples with RIN > 7 were included in the study. Samples were stored at −80°C until sequencing libraries were prepared. Total RNA samples were treated with DNAse I using the Turbo DNA‐free Kit (Thermo Fisher Scientific) according to the kit guidelines. Libraries for RNA sequencing were prepared using the NEBNext Ultra II Directional RNA Library Prep Kit for Illumina with the NEBNext rRNA Depletion Module (New England Biolabs, Ipswich, MA, USA). RNA libraries were sequenced on the HiSeq system (Illumina) for 250 cycles. Sequenced reads were pseudoaligned to hg38 Gencode (v.37) transcriptome using kallisto v.0.48.0 (Bray et al., 2016) with default settings. The average number of reads per sample in the transcriptome analysis was 48.4 (4.8) million. Gene‐level expression abundances were calculated using the tximport Bioconductor package (Soneson et al., 2015). Expression of the *MDM4* gene was presented in transcripts per kilobase million (TPM). Transcriptome analyses of muscle samples from the FUSION and GTEx cohorts were described by Taylor et al. (2019) and the GTEx Consortium (2020).

### 2.6 Evaluation of muscle fibre composition

The muscle fibre composition of the vastus lateralis in athletes was evaluated using immunochemistry, as previously described (Ahmetov et al., 2009). Briefly, vastus lateralis samples were obtained from the left leg using the modified Bergström needle procedure with aspiration under local anaesthesia using 2% lignocaine solution. Serial cross‐sections (7 µm thick) were obtained from frozen samples using an ultratom (Leica Microsystems, Wetzlar, Germany). The sections were then incubated at room temperature in primary antibodies against slow or fast isoforms of the myosin heavy chains (M8421, 1:5000 and M4276, 1:600, respectively; Sigma‐Aldrich, Burlington, MA, USA) for 1 h and incubated in PBS (3 × 5 min). Sections incubated without primary antibodies were to detect non‐specific staining. Images were captured with a fluorescent microscope (Eclipse Ti‐U, Nikon, Tokyo, Japan; Figure S5). The cross‐sectional areas (CSAs) of fast‐ and slow‐twitch muscle fibres were evaluated using ImageJ software (National Institutes of Health, USA). The muscle fibre composition of vastus lateralis (FUSION cohort) and gastrocnemius (GTEx cohort) was estimated based on the expression of the myosin heavy chain 1 (*MYH1*), myosin heavy chain 2 (*MYH2*) and myosin heavy chain 7 (*MYH7*) genes, as previously described (Taylor et al., 2019).

### 2.7 Statistical analysis

Quality control (QC) of the sequencing was performed using the FastQC tool (Andrews, 2010) by Sequencher v.5.4.6 DNA sequence analysis software (Gene Codes Corporation, Ann Arbor, MI, USA) (for details see Supplementary file). After QC, each individual had 18,370 common indel variants to be considered for further analyses. The annotated files were analysed further by Fisher's exact test using PLINK software (Purcell et al., 2007). An exome‐wide association study and principal component analysis were performed using R programming (R Core Team, 2021). Allele frequencies between groups of athletes and control subjects were compared using χ^2^ tests or Fisher's exact test (for small sample sizes). For power athletes (*n* = 31) and control (*n* = 20) groups, exome‐wide associations were evaluated and presented as a Manhattan plot. The mean differences between groups (power vs. endurance athletes) were compared using Student's unpaired *t*‐test. The relationship between gene expression and muscle‐related traits was analysed using regression analysis adjusted for covariates (age and sex). To make scatter plots, the Pearson correlation coefficient was used. All data are presented as the mean (SD). Values of *P* < 0.05 were considered statistically significant.

### Informed consent statement

Informed consent was obtained from all subjects involved in the study.

## 3 RESULTS

**FIGURE 1:**
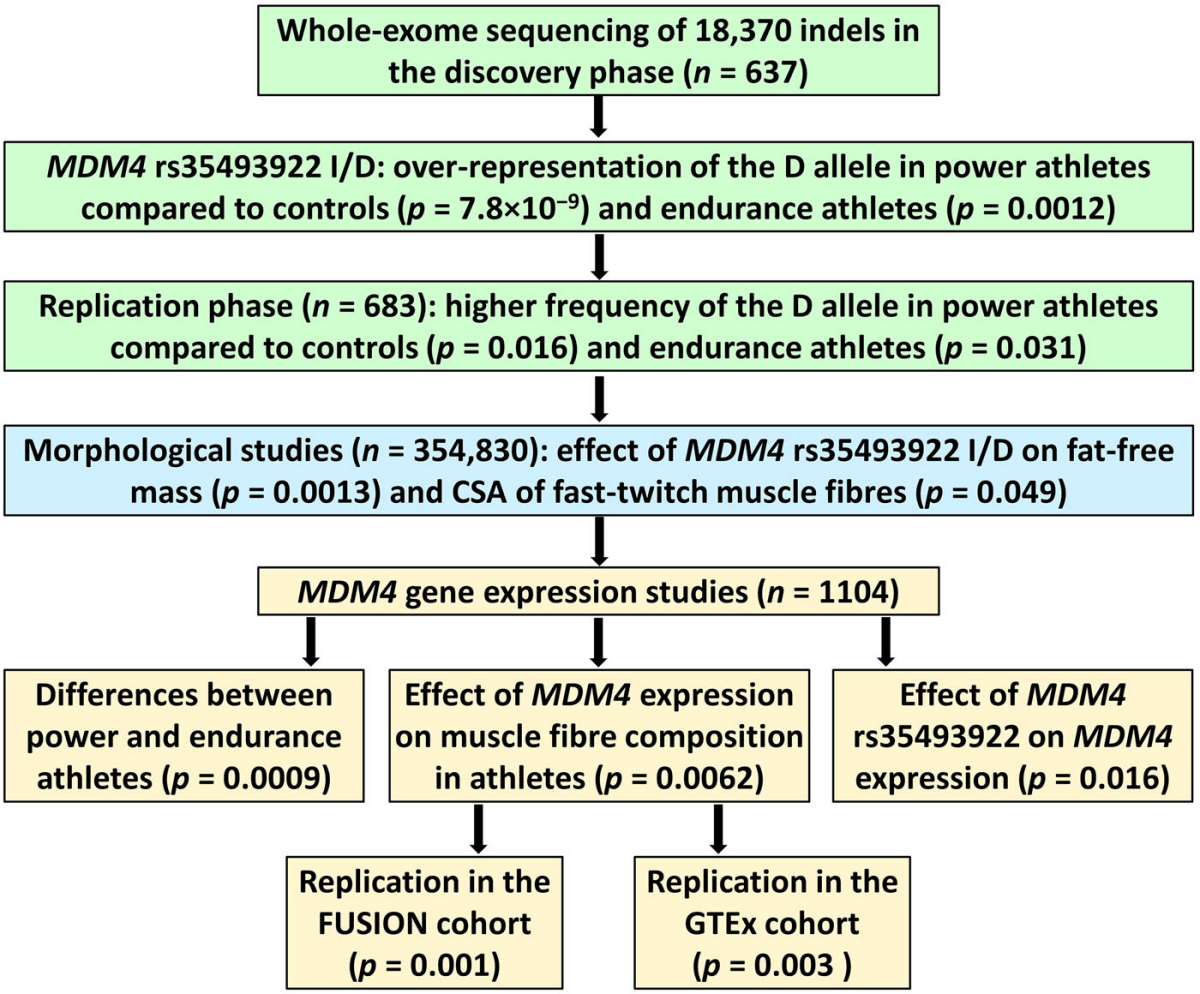
A schematic overview of the study design and main findings. Abbreviation: CSA, cross‐sectional area.

A flow diagram displaying the study design and main findings is shown in Figure 1.

### 3.1 Case–control studies

In the first stage, using a pilot exome‐wide association approach, we compared allelic frequencies of 18,370 indels between 31 Turkish power athletes and 20 control subjects (Figure S6). Three indels, rs35493922, rs34164953 and rs10624210, suggestively (*P* < 1.0 × 10^−5^) deviated between power athletes and control subjects. Next, we compared the allelic frequencies of these three indels between 31 Turkish power athletes and a larger Turkish control group (*n* = 557) from a publicly available database (https://tgd.tuseb.gov.tr/en/; accessed 15 April 2024).

**FIGURE 2:**
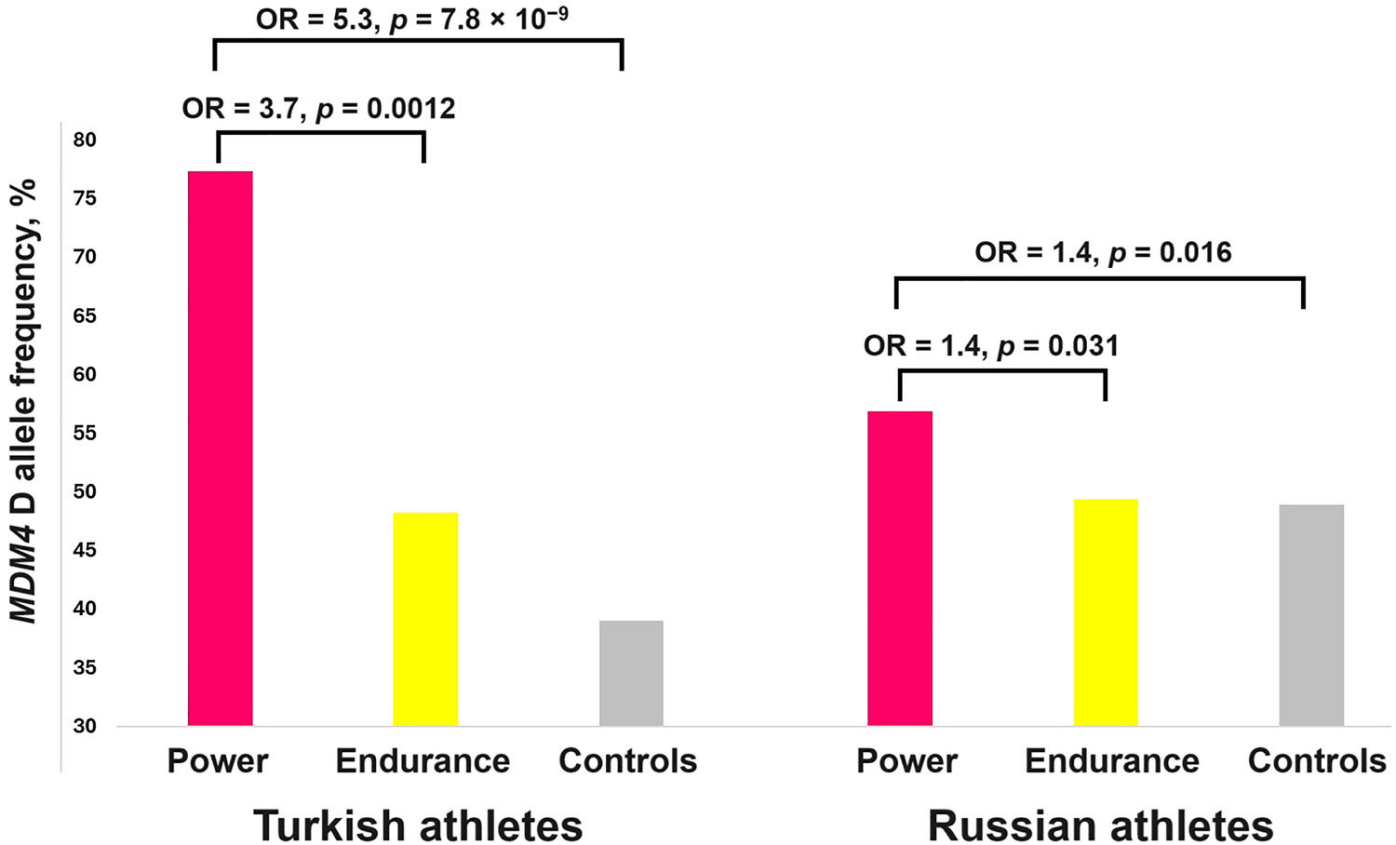
*MDM4* rs35493922 D allele frequency in Turkish and Russian cohorts of athletes and control subjects. Abbreviation: OR, odds ratio.

Of the three indels, only rs35493922, located in the intron of *MDM4*, was associated with power athlete status at a genome‐wide significance level (*P* < 5.0 × 10^−8^). More specifically, the D allele of *MDM4* rs35493922 I/D polymorphism was over‐represented in power athletes (77.4%) compared with control subjects (39.1%; odds ratio = 5.3, *P *= 7.8 × 10^−9^; Figure 2). Furthermore, the D allele was significantly higher in power athletes compared with 29 Turkish endurance athletes (77.4% vs. 48.3%; odds ratio = 3.7, *P *= 0.0012; Figure 2). These findings were replicated in independent cohorts of Russian athletes and control subjects, showing a higher D allele frequency in 305 power athletes (56.9%) compared with 206 control subjects (49.0%; *P *= 0.016) and 172 endurance athletes (49.4%; *P *= 0.031; Figure 2). From the group of Russian power athletes, only sprinters (*n* = 159) had a significantly higher D allele frequency (57.5% vs. 49.0%; odds ratio = 1.4, *P* = 0.027) compared with the control group (Table S1).

### 3.2 Follow‐up studies in the UK Biobank cohort

Analysis of the summary statistics of the UK Biobank data (Open Targets Genetics, 2023) revealed that the *MDM4* rs35493922 D allele was positively associated with greater whole‐body fat‐free mass (*n* = 354,808, β = 0.0476, *P* = 0.0013) and standing height (*n* = 360,388, β = 0.0451, *P* = 0.0025).

### 3.3 Bioinformatic analyses, muscle biopsy and gene‐expression studies

**FIGURE 3:**
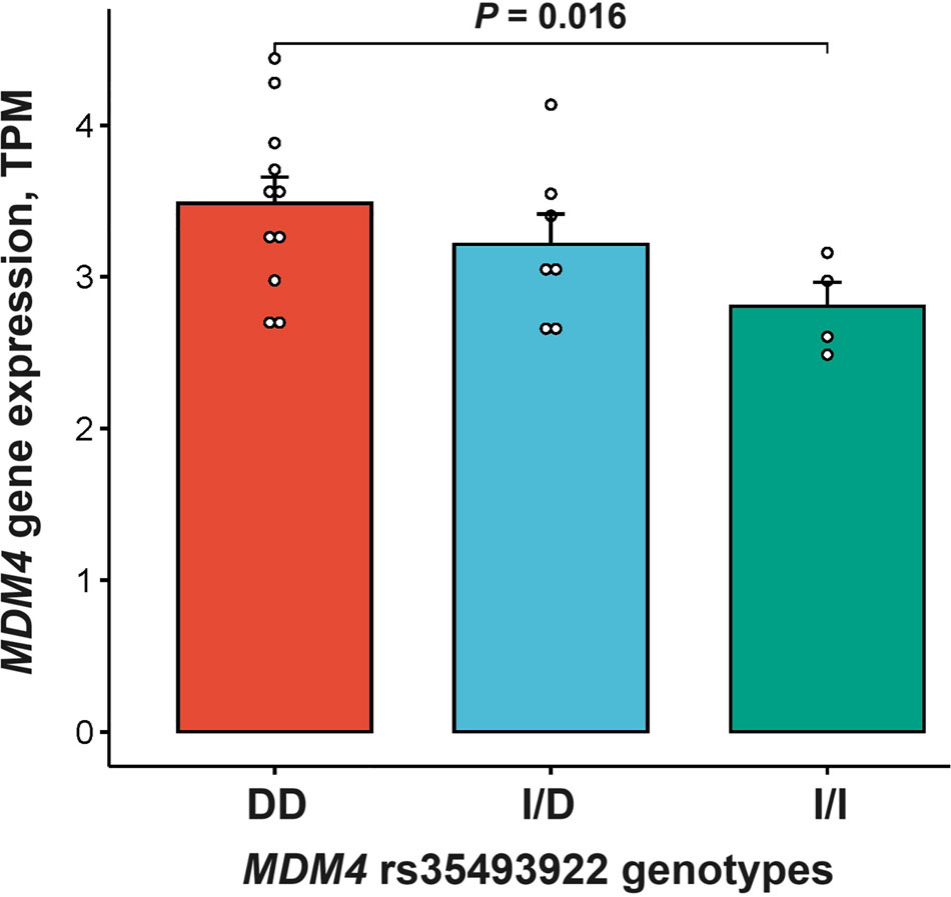
Comparison of *MDM4* gene expression between individuals with different *MDM4* rs35493922 genotypes (*n* = 22).

Bioinformatic analyses revealed that rs35493922 is located in the intronic region of the MDM4 regulator of p53 (*MDM4*) gene. We hypothesized that the discovered rs35493922 I/D (GA/G) polymorphism might be functional and alter *MDM4* gene expression. According to available datasets, the rs35493922 I/D polymorphism was associated with altered *MDM4* gene expression across various tissues. For example, the *MDM4* rs35493922 D allele was associated with higher *MDM4* gene expression in B cells (β = 0.103; *P* = 5.0 × 10^−6^; Fairfax et al., 2012). Furthermore, using muscle samples of the vastus lateralis of 22 males, we found that carriers of the *MDM4* DD genotype had significantly higher expression of *MDM4* in comparison to II homozygotes [3.5 (0.6) vs. 2.8 (0.3) TPM; *P* = 0.016; Figure 3].

**FIGURE 4:**
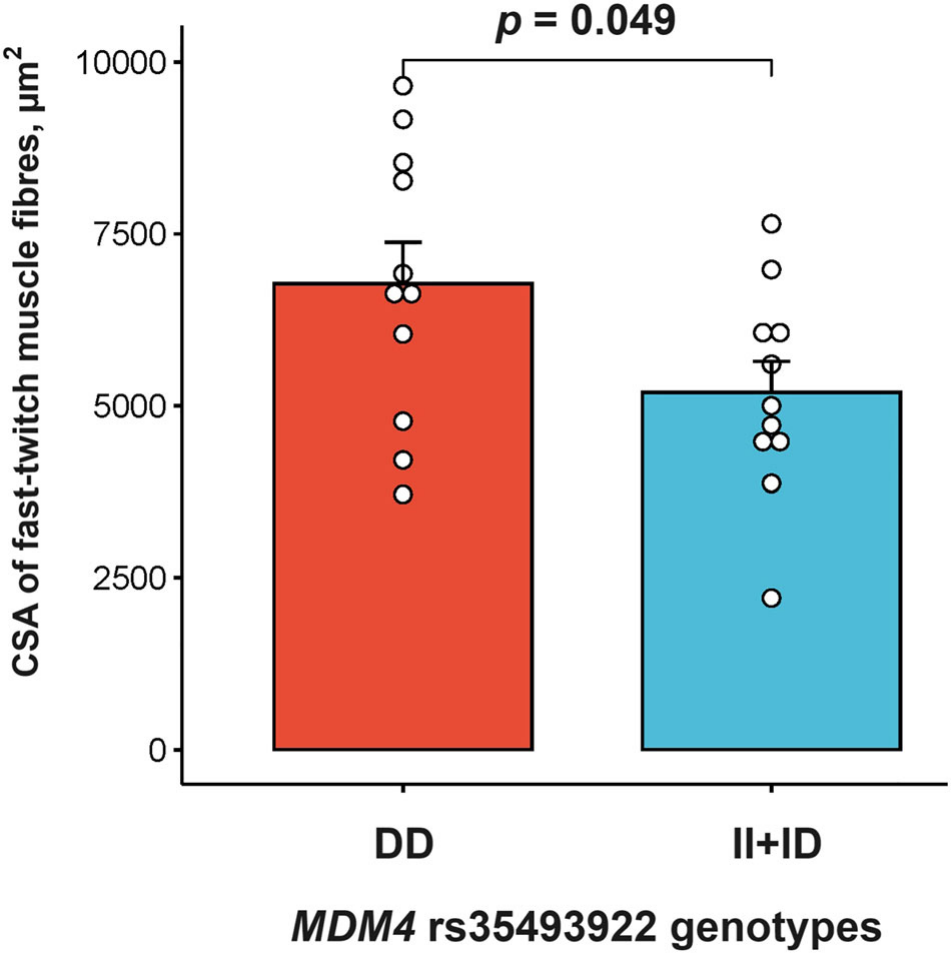
Comparison of cross‐sectional area of fast‐twitch muscle fibres between individuals with different *MDM4* rs35493922 genotypes (*n* = 22). Abbreviation: CSA, cross‐sectional area.

*MDM4* encodes a nuclear protein that inhibits the activity of the p53 tumour suppressor protein, which induces skeletal muscle fibre atrophy. We therefore tested the *MDM4* gene expression in relationship to muscle fibre characteristics. Initially, we found that among 22 males, the *MDM4* DD homozygotes had a significantly higher (*P* = 0.049) CSA of fast‐twitch muscle fibres in comparison to carriers of the I allele [DD, 6777 (1998) µm^2^; II+ID, 5191 (1512) µm^2^; Figure 4].

**FIGURE 5:**
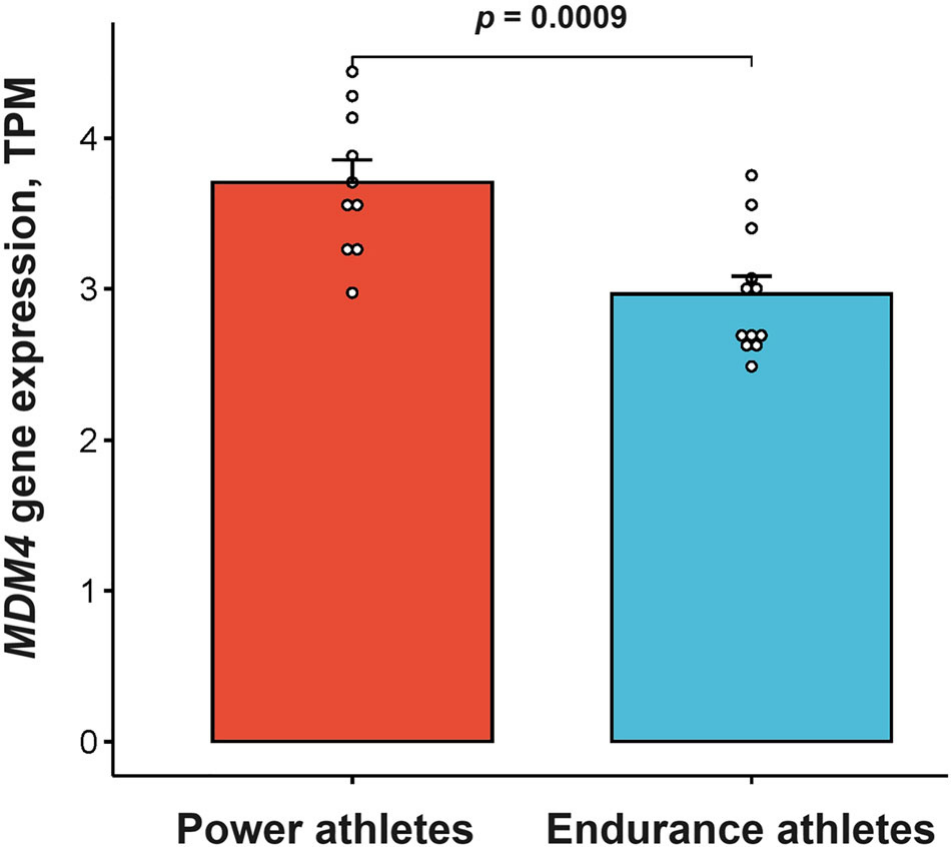
Comparison of *MDM4* gene expression between power and endurance athletes (*n* = 22).

**FIGURE 6:**
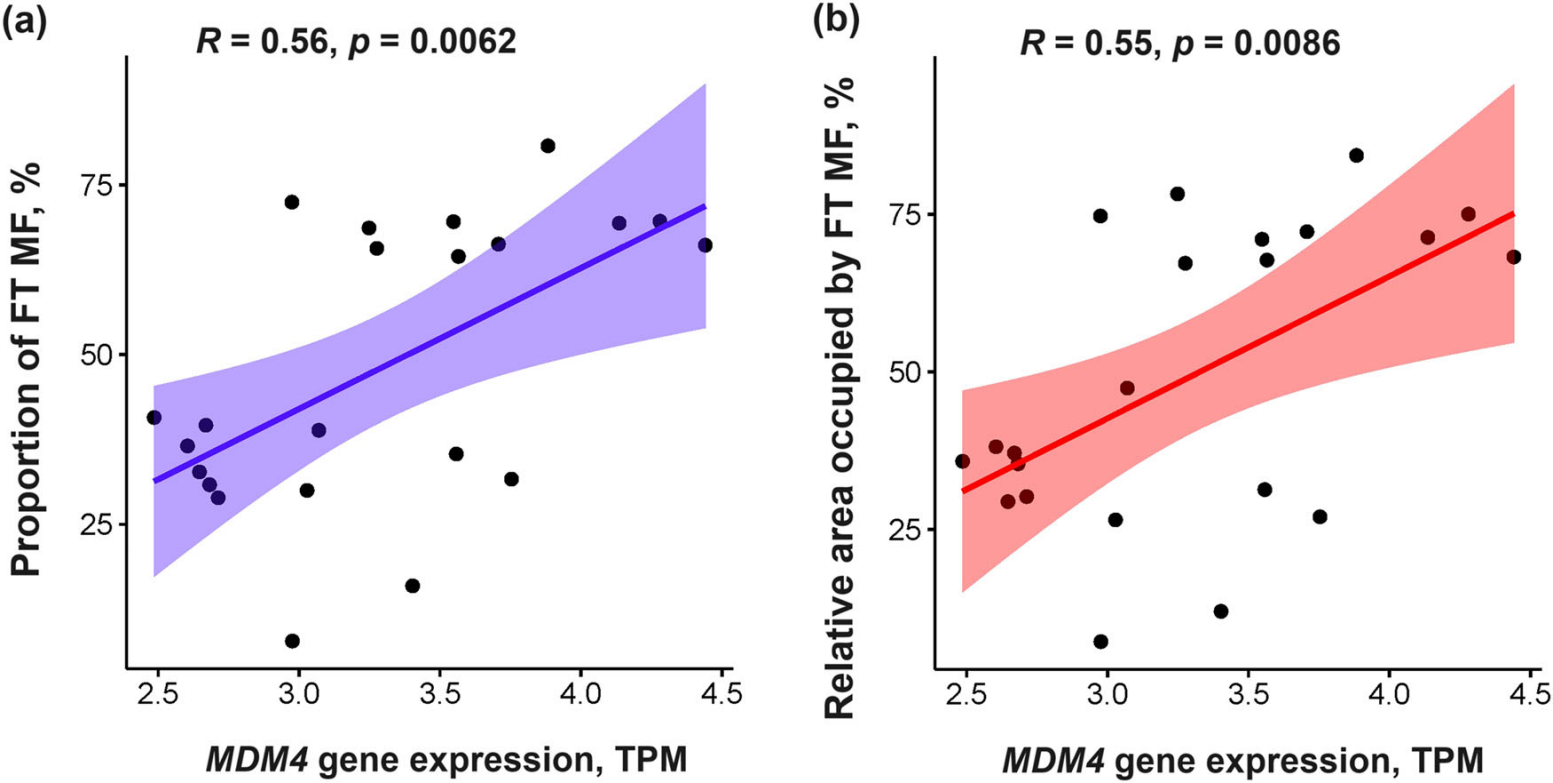
Correlation between *MDM4* gene expression and proportion of fast‐twitch muscle fibres (a), and relative area occupied by fast‐twitch muscle fibres (b) among athletes (*n* = 22). Abbreviation: FT MF, fast‐twitch muscle fibres.

Next, we found that *MDM4* expression was significantly higher in the vastus lateralis of power compared with endurance athletes [3.7 (0.5) vs. 3.0 (0.4) TPM, *P *= 0.0009; Figure 5] and positively correlated with the percentage of fast‐twitch muscle fibres (*r *= 0.56, *P *= 0.0062; Figure 6a) and the relative area occupied by fast‐twitch muscle fibres (*r *= 0.55, *P *= 0.0086; Figure 6b). The association between *MDM4* gene expression and the increased proportion of fast‐twitch muscle fibres was confirmed in the FUSION (vastus lateralis: *n *= 291, *P *= 0.001 adjusted for age and sex) and GTEx (gastrocnemius: *n *= 791, *P *= 0.003 adjusted for age and sex) cohorts.

## 4 DISCUSSION

To the best of our knowledge, this is the first exome‐wide association study with a specific focus on the association of indel variants with power athlete status. We found that the D allele of the *MDM4* gene rs35493922 polymorphism was significantly over‐represented in two independent cohorts of power athletes, including sprinters, in comparison to control subjects and endurance athletes. Furthermore, the *MDM4* D allele and DD genotype were positively associated with higher *MDM4* gene expression, greater whole‐body fat‐free mass and standing height in the UK Biobank cohort, and with greater CSA of fast‐twitch muscle fibres in male athletes. Given that fat‐free mass, height and muscle fibre CSA are positively associated with sprinting, jumping and throwing performance (Guilherme, Semenova, Larin et al., 2022; Methenitis et al., 2016), our findings partly explain the mechanism of the positive selection of the favourable *MDM4* genetic variant (i.e., D allele and DD genotype) in power athletes.

*MDM4* (located on 1q32.1) encodes a nuclear protein that inhibits the activity of the tumour suppressor protein p53 (encoded by *TP53*) through direct binding to its transcriptional activation domain (Popowicz et al., 2008). Given that MDM4 mediates its effect through p53, it would be appropriate to consider the influence of MDM4 on exercise‐ and sport‐related phenotypes inseparably from p53. Accordingly, various stimuli, such as unloading, denervation and ageing, increase the expression of *TP53* and its target genes in skeletal muscle, indicating a significant role of p53 in the development of atrophy (Gorza et al., 2021). Indeed, elevated p53 activity might cause senescence, permanent cell cycle arrest and apoptosis, a well‐known mechanism of cell death (Mijit et al., 2020). Transgenic mice with increased p53 activity develop a wide range of ageing‐associated phenotypes, including skeletal muscle atrophy (Tyner et al., 2002). Muscle fibres deficient in p53 exhibit partial resistance to atrophy caused by immobilization, whereas overexpression of p53 triggers muscle fibre atrophy even without immobilization (Fox et al., 2014). Future studies should test the hypothesis that inhibiting *MDM4* will suppress muscle protein synthesis in growing myotubes, as has been shown in previous experiments with other genes (Baumert et al., 2024).

Furthermore, studies indicate that p53 plays a role during differentiation and development of specific lineages, including skeletal myogenesis (Schwarzkopf et al., 2006). The expression of p53 has varying effects on different muscle fibre types in tumour‐induced cachexia. Specifically, the reduction in fast‐twitch fibre size is significantly less pronounced in p53 null mice. In contrast, the absence of p53 has only a minor impact on slow‐twitch fibres (Schwarzkopf et al., 2006). Interestingly, we found that *MDM4* gene expression is positively associated with the proportion of fast‐twitch muscle fibres in three independent cohorts. Furthermore, we found that the D allele, which is associated with increased expression of *MDM4*, is linked to an increased CSA of fast‐twitch muscle fibres. However, the mechanism of the link between the p53–MDM4 axis and muscle fibre composition is not clear and warrants further investigation involving animal models.

Overall, one might hypothesize that decreased *TP53* gene expression and increased *MDM4* gene expression favour skeletal muscle hypertrophy. Indeed, a previous genome‐wide association study reported that two genetic variants located in *TP53* (3′ untranslated region variant rs78378222 T/G) and *MDM4* (intronic indel rs59912938 C/CT) are associated with appendicular lean mass in the UK Biobank (*n* = 450,243) at a genome‐wide significant level (Pei et al., 2020). More specifically, the *TP53* rs78378222 G allele, which predicts low *TP53* gene expression in blood (*P* = 6.0 × 10^−21^; eQTLGen project), is associated with increased lean mass (*P* = 4.5 × 10^−56^) and greater handgrip strength (*P* = 2.0 × 10^−7^; Open Targets Genetics). Furthermore, the *MDM4* rs59912938 CT allele, which predicts higher *MDM4* gene expression in B cells (*P* = 1.0 × 10^−12^; Fairfax et al., 2012), is associated with increased lean mass (*P* = 3.6 × 10^−16^) and greater handgrip strength (*P* = 0.0000064; Open Targets Genetics). Finally, the *MDM4* rs35493922 D allele, discovered in our study, also predicts increased *MDM4* gene expression in skeletal muscle (*P* = 0.016) and B cells (*P* = 5.0 × 10^−6^; Fairfax et al., 2012), and is associated with increased fat‐free mass (*P *= 0.0013) and greater CSA of fast‐twitch muscle fibres (*P* = 0.049).

The *MDM4* rs35493922 I/D is in strong linkage disequilibrium (*r* ^2^ ≥ 0.94, *D′* ≥ 0.97) with rs4252745, rs10900598 and rs12118859 polymorphisms located in the 3′ untranslated region of *MDM4* (possibly causal variants). Furthermore, the *MDM4* rs35493922 indel might alter regulatory motifs of various transcription factors, including CIZ, Dbx1, Dbx2, Foxj1, Foxp1, HDAC2, HMG, HNF1, Hoxa7, Hoxd8, Isl2, Lhx3, Ncx, Nkx6, Sox, TATA and ZEB1 (HaploReg v.4.2). Transfection studies are needed to show that the *MDM4* D allele has higher transcriptional activity than the common I allele. Furthermore, electrophoretic mobility shift assays are necessary to demonstrate that the I/D polymorphism influences the binding of specific transcription factors. Thus, the mechanisms through which such altered *MDM4* activity influences exercise‐ and sport‐related phenotypes remain speculative, and further in vitro and in vivo studies of gene function are advocated. Nevertheless, we suggest that the D allele of the rs35493922 polymorphism, by increasing the expression of *MDM4*, might lead to decreased p53 activity and prevent skeletal muscles from atrophy, thus making them more susceptible to hypertrophy in response to anabolic stimuli.

Interestingly, MDM4 levels were found to be elevated in response to insulin‐like growth factor 1 (Gilkes et al., 2008). Furthermore, testosterone reduces the activation of p53 (Pronsato & Milanesi, 2016). Overall, literature data and our findings indicate that the p53–MDM4 axis is involved in the regulation of skeletal muscle mass and might be important in athletic performance. It should be noted that previous studies involving genetic markers associated with anabolic factors have also been linked to the power/strength status of athletes (Ahmetov et al., 2014; Gabbasov et al., 2013; Guilherme et al., 2021; Guilherme, Semenova, Borisov et al., 2022; Maciejewska‐Skrendo et al., 2019).

The present study has several limitations that need to be considered when interpreting the results. First, studies evaluating the relationship between genetics and sports performance often face the challenge of recruiting a large and representative sample of athletes. For example, confidentiality and discrimination issues might prevent elite athletes from participating in genetic research or sharing their genetic information with others. This might reduce the statistical reliability and general validity of the findings, in addition to the power to detect small genetic effects. We thus acknowledge that our pilot studies with Turkish athletes (*n* = 60) and muscle samples of athletes (*n* = 22) had a limited impact. It is therefore strongly recommended to perform several replication studies with meta‐analysis (Semenova et al., 2020). Second, the complex and multivariate nature of genetics can make it difficult to determine the clear association of specific genes or gene variants with sporting ability. Different sports require diverse physiological characteristics and abilities, such as endurance, strength, speed, agility and coordination, which might be under the influence and interaction of many genes and environmental factors. Third, although we found a relationship between *MDM4* gene expression and muscle fibre composition in three independent cohorts, we could not establish a molecular mechanism that explains this phenomenon. Furthermore, the role of the p53–MDM4 axis in muscle fibre specification has never been reported before.

## 5 CONCLUSIONS

In conclusion, our data suggest that the *MDM4* rs35493922 deletion variant is positively associated with *MDM4* gene expression, power athlete status, fat‐free mass, CSA of fast‐twitch muscle fibres and height. Furthermore, our gene‐expression analyses consistently show that higher *MDM4* gene expression is positively associated with a greater proportion of fast‐twitch muscle fibres in three independent cohorts.

## AUTHOR CONTRIBUTIONS

Conceptualization: Celal Bulgay, Anıl Kasakolu, Ildus I. Ahmetov and Hasan H. Kazan. Formal analysis: Anıl Kasakolu, Seyrani Koncagul, Hasan H. Kazan, George John, Ekaterina A. Semenova, Rinat I. Sultanov, Rinat A. Yusupov and Ildus I. Ahmetov. Investigation: Celal Bulgay, Hasan H. Kazan, Anıl Kasakolu, Seyrani Koncagul, George John, Rinat I. Sultanov, Andrey V. Zhelankin, Nikolay A. Kulemin, Andrey K. Larin, Edward V. Generozov and Mehmet A. Ergun. Writing—original draft preparation: Celal Bulgay, Hasan H. Kazan, Anıl Kasakolu, Seyrani Koncagul, Mehmet A. Ergun and Ildus I. Ahmetov. All authors approved the final version of the manuscript and agree to be accountable for all aspects of the work in ensuring that questions related to the accuracy or integrity of any part of the work are appropriately investigated and resolved. All persons designated as authors qualify for authorship, and all those who qualify for authorship are listed.

## CONFLICT OF INTEREST

The authors declare no conflict of interest.

## Supporting information

Supporting Information.

## Data Availability

The exome‐wide association study dataset is available online at: https://doi.org/10.6084/m9.figshare.24496216.v1 (accessed 10 June 2024). The human data (fat‐free mass; muscle biopsies) are available online at: https://genetics.opentargets.org/Variant/1_204536848_GA_G/ (accessed 10 June 2024) and in the dbGaP database (phs000424.vN.pN; phs001048.v2.p1; accessed 10 June 2024).
