# Supplementary material for: Association analysis of indel variants and gene expression identifies MDM4 as a novel locus for skeletal muscle hypertrophy and power athlete status
Source: Exp Physiol. 2024 Jul 23;110(11):1661–71. doi: 10.1113/EP091992 (PMC12575997; doi:10.1113/EP091992)
Supplement: Supplementary file 1 — Supporting Information. [file EPH-110-1661-s001.docx]

Supplementary file

Association analysis of indel variants and gene expression identifies *MDM4* as a novel locus for skeletal muscle hypertrophy and power athlete status

**1. Supplementary Methods**

**1.1. Quality control (QC) of the sequencing**

Quality control (QC) of the sequencing was performed using the FastQC tool (Andrews, 2010) by Sequencher v5.4.6 DNA sequence analysis software (Gene Codes Corporation, Ann Arbor, MI USA).

*An example quality control (QC) report of fastq*

The QC of one of the samples (sample 1, having two fastq files created for forward and reverse reading) was assessed using the FastQC tool (Andrews, 2010) by Sequencher v5.4.6 DNA sequence analysis software (Gene Codes Corporation, Ann Arbor, MI USA). According to the results, the total number of sequences was 30,037,433, the sequence lengths were 35-151 bp (Figure S1), the quality scores across all bases were approximately 35 (Figure S2), the Phred scores for mean sequence quality were 35 (Figure S3), and the GC% content was 51% (Figure S4).


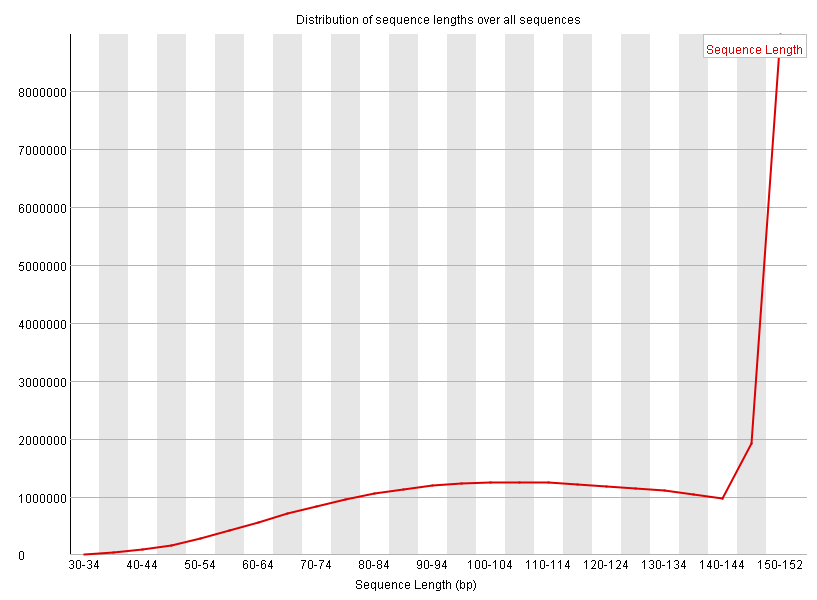


Figure S1. Sequence length distribution.


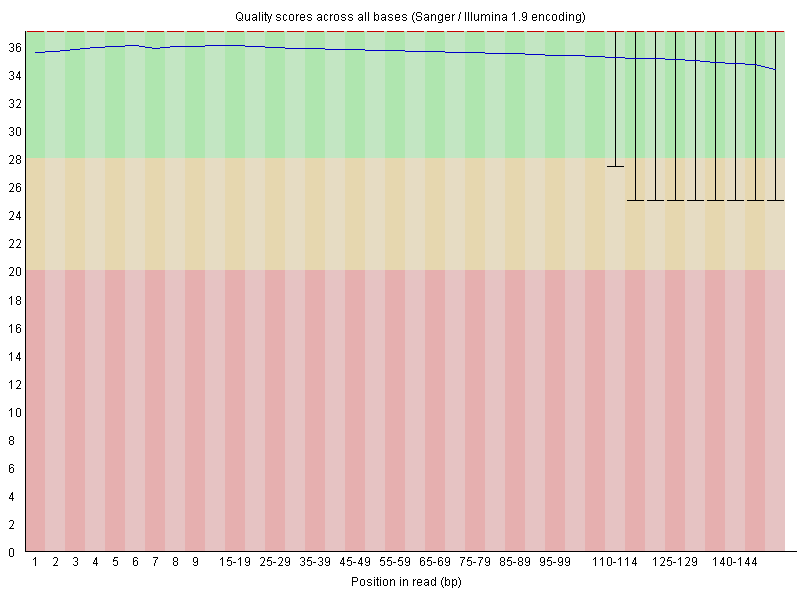


Figure S2. Per base sequence quality.


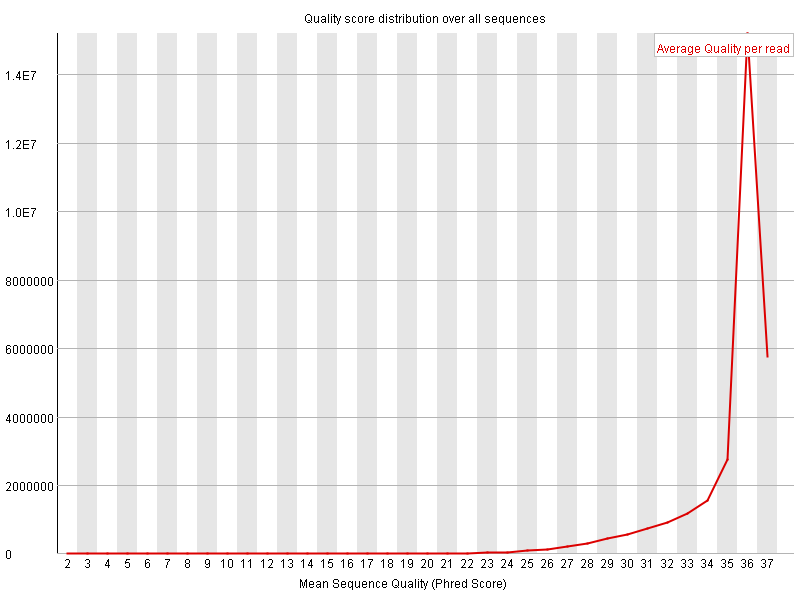


Figure S3. Per sequence quality scores.


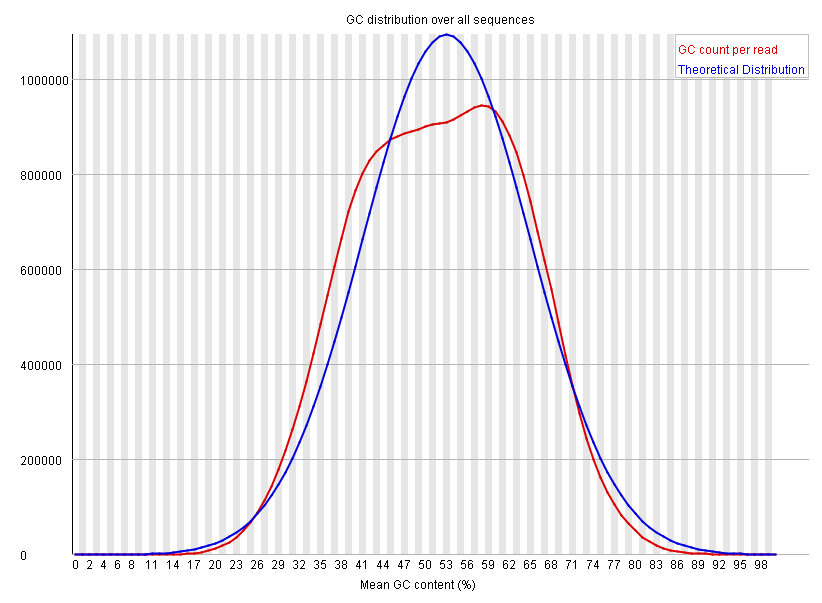


**Figure S4.** Per sequence GC content.

The dataset comprised a total of 50,868 indel variants, precisely located within the hg38 reference genome. This dataset was extracted utilizing the VariantAnnotation package within the Bioconductor framework from a VCF file. QC workflow was as follows:

1. Indel Call Rate Assessment: <0.90
2. Genotype (individual) Call Rate Assessment: <0.90
3. Minor Allele Frequency Assessment: <0.05

**1.2. Evaluation of muscle fiber composition with immunochistochemistry**

**
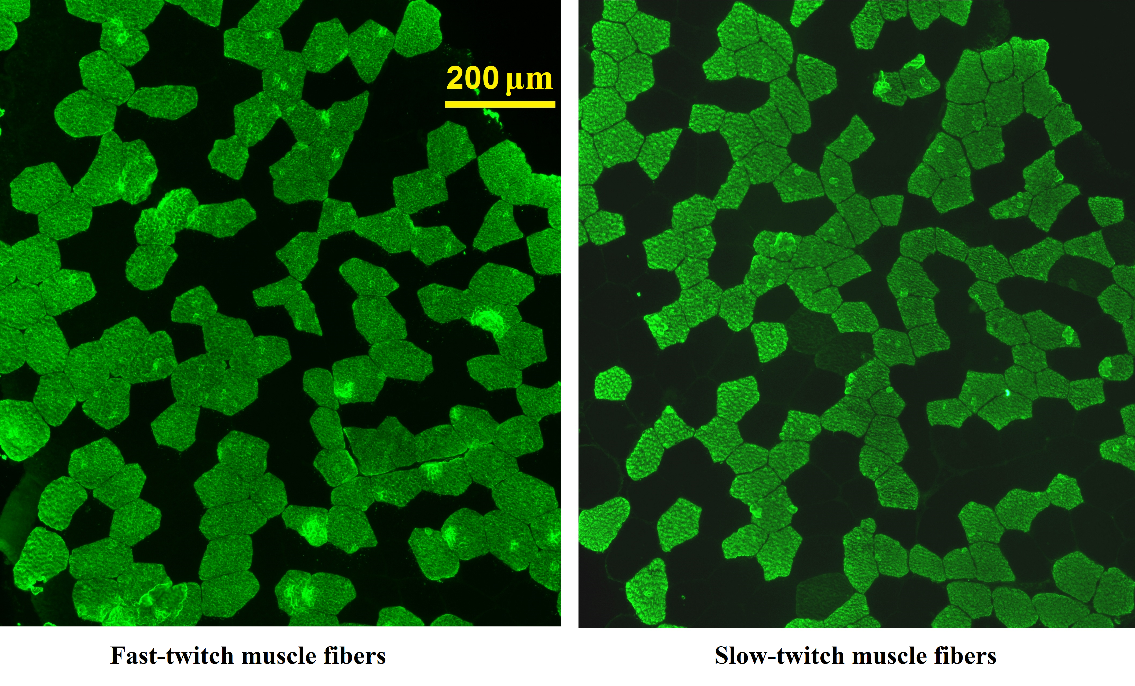
**

**Figure S5.** Microphotographs of the labelled muscle sections (immunohistochemistry)

**2. Supplementary Results**


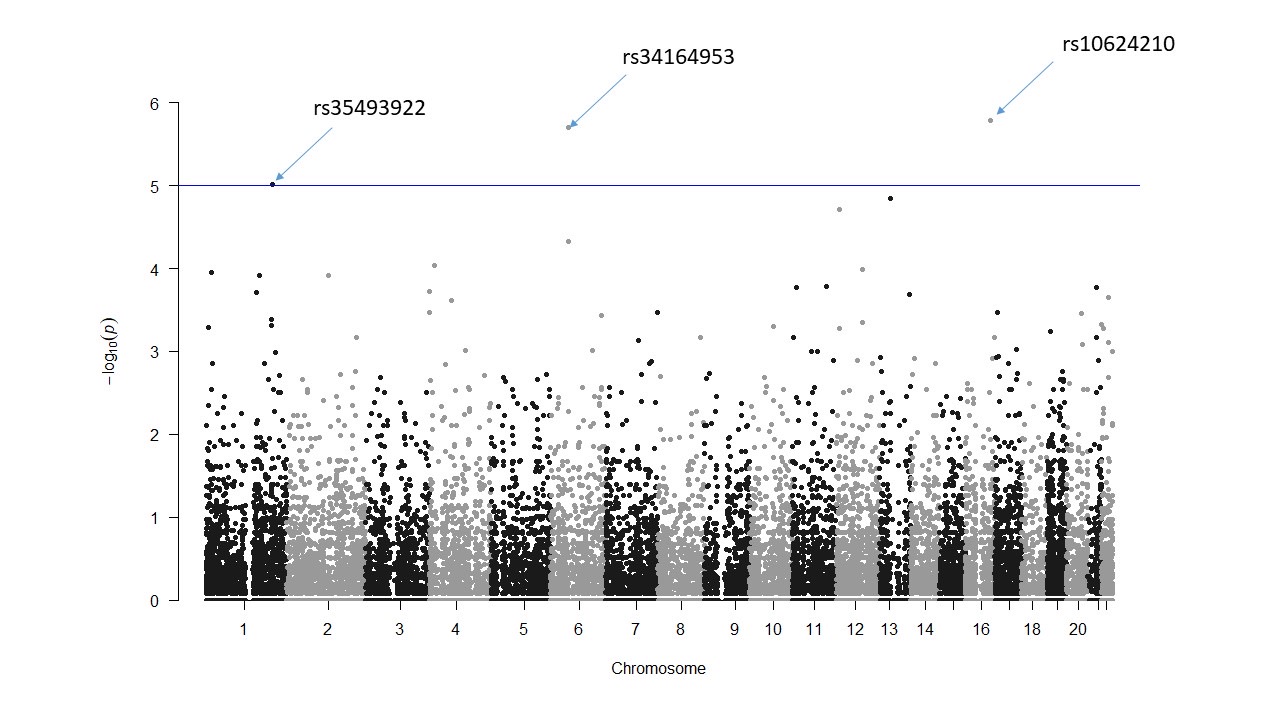


**Figure S6.** Manhattan plot showing associations between indels across chromosomes and power athlete status (comparison between 31 power athletes and 20 controls). Each dot points to an indel localized on a related chromosome given on the x-axis, while the y-axis gives the -logp values for the related indel association. Light or dark blue was used for the discrimination of the chromosomes in the figure. Three indels, rs35493922, rs34164953, and rs10624210, suggestively (*p* < 1.0×10^–5^) deviated between power athletes and controls.

**Table S1.** *MDM4* rs35493922 genotypes distribution and D allele frequency in athletes and controls from two independent cohorts.

| **Groups** | **n** | **II** | **ID** | **DD** | **D, %** | ***P* value (power vs controls)** | ***P* value (power vs endurance)** |
| --- | --- | --- | --- | --- | --- | --- | --- |
| Turkish power athletes | 31 | 1 | 12 | 18 | 77.4 | 7.8×10^−9^* | 0.0012* |
| Turkish endurance athletes | 29 | 8 | 14 | 7 | 48.3 | ─ | ─ |
| Turkish controls** | 557 | N/A | N/A | N/A | 39.1 | ─ | ─ |
|  |  |  |  |  |  |  |  |
| Russian sprinters | 159 | 26 | 83 | 50 | 57.5 | 0.027* | 0.044* |
| Russian speed-strength athletes | 57 | 10 | 33 | 14 | 53.5 | 0.459 | 0.516 |
| Russian strength athletes | 89 | 20 | 35 | 34 | 57.9 | 0.06 | 0.082 |
| All Russian power athletes | 305 | 56 | 151 | 98 | 56,9 | 0.016* | 0.031* |
| Russian endurance athletes | 172 | 42 | 90 | 40 | 49.4 | ─ | ─ |
| Russian controls | 206 | 54 | 102 | 50 | 49.0 | ─ | ─ |

**p*<0.05, statistically significant differences

** <https://tgd.tuseb.gov.tr/en/variant/1-204536848-GA-G>

**References**

Andrews, S. (2010). FastQC: A Quality Control Tool for High Throughput Sequence Data [Online]. Available online at: <http://www.bioinformatics.babraham.ac.uk/projects/fastqc/>
